# The Modulatory Effect of Acupuncture on the Activity of Locus Coeruleus Neuronal Cells: A Review

**DOI:** 10.1155/2017/9785345

**Published:** 2017-10-18

**Authors:** Gihyun Lee, Woojin Kim

**Affiliations:** Department of Physiology, College of Korean Medicine, Kyung Hee University, Seoul 02447, Republic of Korea

## Abstract

The Locus Coeruleus (LC) is a small collection of noradrenergic neurons located in the pons. In the brain, noradrenaline (NE) is primarily produced by noradrenergic cell groups in the LC, which is the largest group of noradrenergic neurons in the central nervous system. Acupuncture, including the electroacupuncture which is a modified acupuncture method, is known to be effective in various kinds of diseases, and the involvement of noradrenergic system in the central nervous system has been reported by previous studies. However, on whether acupuncture can modulate the LC neuronal cells activities, results vary from studies to studies. In this paper, we included twelve articles, which observed the effect of acupuncture on the activities of LC in humans and animals. Our study shows that, among twelve included studies, six reported decrease of LC activities, whereas six showed increase of LC activities after acupuncture treatment. Although it is difficult to draw a firm conclusion, the authors suggest that the difference of frequencies may play an important role in the modulatory effect of acupuncture on LC. Further studies are needed to clarify the precise mechanism of acupuncture on LC, as it can lead to a new therapeutic method for various LC-NE related diseases.

## 1. Introduction

The Locus Coeruleus (LC), meaning the blue spot in Latin, is a small collection of noradrenergic neurons (about 16,000 per hemisphere in the human), located just behind the periaqueductal gray (PAG) in the dorsorostral pons [1]. In the brain, noradrenaline (NE) is primarily produced by noradrenergic cell groups classified as A1–A7, which projects NE to widespread area of the brain. Among them, A5–A7 groups project not only to the brain but also to the spinal cord [2], and the A6, which is the LC, is the largest group of noradrenergic neurons in the central nervous system (CNS) [3]. NE, as other catecholamines, dopamine, and epinephrine, possesses two hydroxyl groups and one amine group bound to a benzene ring, and it is biosynthesized from tyrosine. Tyrosine is first converted into dopamine, and dopamine is further converted into NE by dopamine-beta-hydroxylase (DBH) present in noradrenergic cells [4].

NE exerts its effect in various parts of the CNS by its receptors present on cells. The most widely known noradrenergic receptors are *α*1-, *α*2-, or *β*-adrenoceptors. Activation of *α*1-adrenoceptors and *β*-adrenoceptors by NE is reported to generally excite the follower cells [5, 6]. In contrast, activation of *α*2-adrenoceptors was demonstrated to inhibit the follower cells [5]. *α*2-Adrenoceptors are widely distributed in the spinal cord as well as in the brain [7]. Via these receptors, NE produced in LC exerts many different functions in our body, and their function is known as the LC-NA system. The LC-NA system was reported to be important in learning and memory [8] and sleep-wake cycle [6, 9]. Also, by stimulating the sympathetic nerve, they could regulate the blood pressure [10] and play an important role in the stress [11]. Furthermore, they are also closely related to pain [4], as NE is known to inhibit the transmission of the pain, by acting through the *α*2-adrenoceptors present at the spinal cord, at the pre- and postsynaptic neurons. As one of the major NE producing sites in the brain, LC along with the PAG and rostral ventromedial medulla (RVM) play an important role in pain modulation, as they are known to produce NE, endogenous opioid, and serotonin, respectively. These neurotransmitters are involved in pain inhibition by acting through their receptors at the spinal cord [12].

Acupuncture is a treatment method that has a long history, and nowadays acupuncture along with electroacupuncture, which is a modified method by providing electrical current through acupuncture, is used throughout the world. According to the report released by the World Health Organization (WHO), more than 40 disorders, including stress and insomnia, can benefit from acupuncture treatment [13]. Furthermore, it is recommended for various types of pain, such as low back pain [14], knee pain [15], and headache [16]. The analgesic effect of acupuncture is widely known, and, by numerous studies conducted both in humans and in rodents [17, 18], its effect has been proven. Studies performed on animals have shown that acupuncture can significantly relieve behavioral signs such as hyperalgesia and allodynia in peripheral nerve injury-induced neuropathic pain models [19–21]. Also, it was reported to reduce hypertension [22] and stress [23]. However, although the curative effect of acupuncture is continuously reported by clinical and experimental studies, the mechanism that lies behind it is not fully understood, especially in the brain.

Over the last several decades, researchers have clarified the involvement of NE in the action of acupuncture [24], by using diverse animal models. In pain, by reporting that the action of acupuncture was blocked by *α*2-adrenoceptors antagonist yohimbine, they demonstrated the involvement of NE in acupuncture analgesic mechanism [19]. In accordance with these results, many articles mentioned the LC as the source of the NE action in the spinal cord after acupuncture treatment [19, 21, 24]. However, results of experiments conducted to clarify the effect of acupuncture in the LC were not consistent. Some reported that LC activities were increased by acupuncture treatment, whereas some reported that they were decreased by acupuncture treatment. Thus, in this review, we will first state the differences of experimental methods and the results of all twelve included papers, and, in the discussion, we will try to analyze the results and will further try to draw a conclusion. Given the importance of LC-NA system in our body and the implication of acupuncture in NE system, we believe that a timely review is important, to guide future efforts in the advancement of acupuncture treatment, as well as in the acupuncture related researches. Based on the previously published studies, we will proceed to expand on clarification of the effect of acupuncture on the activity of the LC neuronal cells.

## 2. Modulation of LC Neuronal Cells by Acupuncture

In our review, we have included twelve articles which analyzed the effect of acupuncture on LC neuronal cells. From the twelve included studies, one was assessed in humans, whereas others were assessed in animals such as rats, rabbits, and goats. Most articles used ST36 acupoint; however, other acupoints such as GB30 and LI4 were also used. The stimulation frequency and duration were also different, as well as the methods they used to assess the effect on the LC. Studies conducted in human used functional magnetic resonance imaging (fMRI), and most studies conducted with animals used c-Fos expression method. The results were also different. Among twelve studies, six reported the decrease of LC activities following acupuncture stimulation, whereas six reported the increase of LC activities after EA treatment (Table 1). Thus, in this part, we will divide articles into two parts: increased and decreased activity of LC after acupuncture treatment.

### 2.1. Increased LC Activity by Acupuncture

To assess whether the low (4 Hz) or high (100 Hz) frequencies of EA administered at ST36 could affect the number of Fos-Like Immunoreactive (FLI) neurons in the LC and in the spinal cord, Lee and Beitz [25] used lightly anesthetized rats. For control, acupuncture was administered at ST36 without any electrical stimulation. Their results show that three hours of both low and high frequency of EA treatment exhibited a significantly greater number of Fos-labeled neurons in the dorsal horn of the L2 spinal cord segment and the LC.

Kwon et al. [26] also demonstrated the effect of EA by using similar protocol to Lee and Beitz, as low (4 Hz) or high (100 Hz) frequencies of EA were used to see the cellular activity of central catecholaminergic (CA) synthesizing neurons in the LC. Immunohistochemistry with double labeling method between FLI neurons and DBH- or tyrosine hydroxylase- (TH-) positive neurons was used, as TH-positive neurons are an indicator of CA. They used naïve rats and anesthetized the animals with isoflurane throughout the acupuncture treatment. For EA administration, bilateral ST36 acupoints were stimulated for 120 min. Immunohistochemistry was conducted two hours after the electrical stimulation. Their results demonstrate that both frequencies of EA increased the number of FLI neurons in the LC as well as other parts of the brain such as the dorsal raphe (DR), hypothalamic arcuate nucleus (Arc), A5, and A7. Furthermore, both the low and high frequencies of EA increased the number of FLI neurons and the cellular activities of DBH/TH-positive neurons in the LC. Although both the low and high frequencies induced significant increase in the LC, high frequency had a stronger effect compared to low frequency (*p* < 0.01).

Before observing the change in the LC induced by EA, Medeiros et al. used repeated immobilization protocols to exclude the effect of anesthesia or acute immobilization stress on the c-Fos expression. Firstly, they observed the change in brain c-Fos expression following repeated immobilization for two hours/day for 13 days. They found that this method was effective as the c-Fos expression was significantly different from rats which did not undergo repeated immobilization (*p* < 0.001). The 60 min bilateral stimulation of EA at ST36 significantly increased the level of c-Fos expression in the LC compared to the control, where the acupuncture was administered 5 mm lateral to the midline of the posterior surface of the hind limb (*p* < 0.05). However, the effect was not significant in the group where rats did not undergo repeated immobilization. The EA neither increased nor decreased the activity of the LC.

Li et al. [27] have observed the effect of EA on LC by using inflammatory pain rat model. Inflammatory pain was induced by injecting complete Freund's adjuvant (CFA) subcutaneously into one hind paw of rats. For EA treatment, GB30 acupoint was chosen, and GB30 was stimulated for 20 min. For control, acupuncture needles were inserted bilaterally into GB30 without electrical or manual stimulation. Paw withdrawal latency to a noxious thermal stimulus was measured before and after 20 min of EA treatment. Compared to sham EA, EA significantly (*p* < 0.05) increased withdrawal latency of the inflamed hind paws in the sham-operated rats. EA, compared to the sham EA, also significantly inhibited c-Fos expression in laminae I-II of the spinal cord (58.4 ± 6.5 versus 35.2 ± 5.4 per section). However, EA activated serotonin- and catecholamine-containing neurons in the nucleus raphe magnus (NRM) and LC that project to the spinal cord.

Qiu et al. [28] used two-year-old healthy hybrid male goats to evaluate the levels of c-Fos and c-Jun expression induced by EA in the brain. EA were administered at a set of GV20, Santai, Ergen (AH1), and Sanyangluo (TE8), and four different frequencies, such as 0, 2, 60, and 100 Hz, were used. The results show that three frequencies (2, 60, and 100 Hz) of EA induced significant increase of c-Fos and c-Jun expression in the LC, whereas 0 Hz did not induce any significant change in the LC compared to the blank control. Also, the expressions of c-Fos or c-Jun in the LC were not significantly different among the frequencies. However, pain threshold measured by using the potassium iontophoresis test showed that the pain threshold induced by 60 Hz was higher (*p* < 0.01) than that induced by 0, 2, or 100 Hz.

Hu et al. [29] also used healthy crossbred male goats to assess the effect of EA on pain threshold and LC. EA was administered at both GV20 and Santai acupoints for 30 min. Firstly, by measuring the pain threshold using iontophoresis method, they showed that the pain threshold induced by EA at set of GV20-Santai acupoints was 44.74% ± 4.56% higher than that by EA at set of ST36 acupoints (32.64% ± 5.04%). Furthermore, they reported that, compared with blank control, EA at two sets of acupoints increased c-Fos expression in the LC (9.31 ± 2.39 versus 15.27 ± 2.40; *p* < 0.05).

### 2.2. Decreased LC Activity by EA

Napadow et al. [30] observed the effect of EA in humans. Ten healthy volunteers' brains were analyzed by using noninvasive fMRI. For treatment, EA was administered unilaterally at ST36 and was stimulated for 30 min, whereas, for sham acupuncture, the same electrical stimulation was given to a nonacupoint site, 8 cm above the proximal edge of the patella, on the midline of the thigh. The difference of electrical current in the EA and the control group was not significant. They stated that LC activity was decreased after acupuncture treatment. However, the activity of PAG increased after EA treatment.

Cao et al. [31] conducted studies both in humans and in rabbits. In human, they checked the change related to sympathetic nervous system functions such as palm temperature, pulse rate, and pain tolerance threshold. Using rabbits, they conducted biochemical studies. They observed whether EA could elevate or decrease NE level in the LC, PAG, and NRM and in the dorsal horn of the spinal cord by using perfusate method. The baseline of NE level in perfusate of LC was 156 ± 29 pg/ml; however, after 20 min of EA at LI4 and SJ05 points, it dropped markedly to 59.41 ± 7.8 pg/ml (*p* < 0.02). At the same time, the pain threshold to stimulation increased from 0.36 ± 0,09 mA to 1.36 ± 0.22 mA (*p* < 0.001). The NE level also decreased in PAG and NRM areas. However, at the dorsal horn of the spinal cord, the NE level was increased compared to the level of before administrating EA (*p* < 0.02). Cao et al. supposed that EA cause a central inhibition, by decreasing the NE released in both the brain and the plasma, but increasing the NE level in the spinal cord, and suppose that different pathway of NE is involved in the effect of EA.

S. Wang and X. Wang [32] observed the effect of 15 min EA stimulation in rats with hyperactivated bladder induced by intraperitoneally (i.p.) injected L-dopa. Injection of L-dopa induced a frequent micturition and increased basal bladder pressure compared to normal saline injected group in rats. However, this increased frequency and pressure were decreased by bilateral B29 treatment for 30 min. Furthermore, EA decreased the upregulated feedback in the synthesis of DBH in the LC, which resulted in the decreased release of NE from LC, after L-dopa injection. In the L-dopa group, DBH significantly increased to 10.7 ± 1.64 and 15.8 ± 1.28 for 8 and 24 h after the injection, respectively, compared with the normal saline injected control group (*p* < 0.05 each). EA treatment at B29 had an inhibitory effect on L-dopa-caused feedback increase in the synthesis of DBH. In the EA group, DBH significantly decreased to 6.9 ± 1.02, 24 h after a L-dopa injection followed by 15 min after EA treatment as compared with that in the drug group (*p* < 0.05).

Yang et al. reported that EA at Shaohai and Neiguan (HT3-PC6) points significantly attenuated stress induced peripheral responses, such as increased blood pressure, heart rate, and plasma CA [33]. In a subsequent study [23], they reported that 180 min of immobilization stress significantly increased FLI in various areas of the brain related to stress, such as the paraventricular hypothalamic nucleus (PVN), arcuate nucleus (ARN), supraoptic nucleus (SON), and the LC. In their study, bilateral combination stimulation of HT3 and PC6 for 30 min attenuated the increased number of FLI neurons induced by 180 min of immobilization stress in the LC compared to control group (20.5 ± 0.8 versus 15.8 ± 1.0; *p* < 0.01).

Wang et al. examined the effects of acupuncture stimulation to the sacral segment on electroencephalograms (EEGs) and activity of LC neurons in urethane-anesthetized rats [34]. A fine acupuncture needle (diameter, 0.35 mm) was positioned at the periosteum of the sacral segment (S1–S4) by palpation and was rotated manually 1.0–1.5 turns/s for 1 min. Among the four sacral segments, the stimulation of acupuncture was more effective in S3 segments. This acupuncture treatment decreased the frequency of the wave from small-amplitude faster waves to large-amplitude slow waves, which demonstrate that acupuncture stimulation may change the state from light anesthesia to deep anesthesia, and also decreased the activity of noradrenergic neurons in the LC.

Park et al. [35] experimented with rats undergoing immobilization stress for six hours a day for 21 consecutive days. The stress increased the response of the anxiety-related behavior, and the serum level of corticosterone and the number of TH-immunoreactive cells in the LC were also increased. 10 minutes of EA treatment to the ST36 decreased the anxiety-related behavioral response, compared with the stress group. Moreover, the serum corticosterone level and TH-immunoreactive expression were also decreased in the ST36 group, compared to the group treated to the nonacupuncture point in the tail. The anxiety-related behavior was tested using the elevated plus maze and the Vogel test on day 22. The serum concentration of corticosterone was determined using an enzyme-linked immunosorbent assay kit. The expression of TH in the LC was measured by immunohistochemistry.

## 3. Discussion

For more than a thousand years, acupuncture has been used as a treatment method. Through numerous clinical [36, 37] and experimental studies [38, 39], its therapeutic effect has been proven in various spectrums of diseases. However, compared to the large number of studies which investigated on its therapeutic effect, studies to clarify its mechanism in the brain are still relatively small in numbers [40]. Particularly, in the LC, not only are the published experimental studies small, but also no review paper has ever been written, and to our knowledge this is the first review paper.

Over the last several decades, researchers have supposed that acupuncture not only acts at the spinal level but also acts at the supraspinal level, involving the activation of different brain areas. By using pain animal models, researchers have suggested that acupuncture could decrease different types of acute and chronic pain through the descending inhibitory pathway, involving brain areas such as PAG, RVM, and thalamus [21]. LC, as the largest NE producing part in the CNS, was also suggested to be related to acupuncture mediated pain inhibition, as the acupuncture analgesic effect was blocked by *α*2-adrenergic receptors antagonist at the spinal level [19]. Also, by using animal stress model, it was reported that the acupuncture could decrease stress by modulating the NE level of our body [31]. However, although, in the spinal cord and in the peripheral site, various studies showed that NE is playing an important role in the effect of acupuncture, the effect of acupuncture on the activities of the LC neuronal cells is still not clear [24, 41].

In our review also, all included twelve articles show different results. LC activities were found to decrease after acupuncture administration in some studies, whereas, in other studies, the activities of LC increased as demonstrated in Table 1. Among our twelve included studies (Table 1), one study observed the effect of acupuncture on humans [30], whereas eleven observed its effect on various animals. Eight studies used rats, one used rabbits [31], and two used goats [29]. Furthermore, the animal model they used were all different; seven studies assessed the effect on healthy naïve human and animals [26, 29–31, 34], three used immobilization stress to decrease [42] or increase [23, 35] the activity of LC before acupuncture treatment, one used L-dopa induced hyperactivated bladder model [32],and finally one study used CFA-induced inflammatory pain model [27]. Also, the assessment methods were all different. Most studies observed the change of c-Fos expression; however, Wang et al. and Napadow et al. used electrophysiology and fMRI, respectively.

With all the differences of the results and the implicated treatment methods involved in the studies, it is difficult to conclude whether the acupuncture decrease or increase the activity of the LC. However, it may be speculated that the frequency used in acupuncture stimulation may play an important part in the effect of acupuncture. Most studies in which the effect of acupuncture on LC induced the decrease of LC activities used low frequency (<4 Hz) with lesser than 30 minutes of stimulation, whereas most studies, where the acupuncture induced an augmentation of LC activity, used relatively high frequency (≥10 Hz) and longer stimulation duration. By previously published studies conducted on pain, it was shown that the frequency of acupuncture stimulation involved different mechanism in our body. For example, low frequency of acupuncture induced analgesia was abolished following lesions of the arcuate nuclei but not high frequency, whereas selective lesions of the parabrachial nuclei attenuated high frequency induced analgesia but not low frequency [43]. These results stated that the mechanism of low and high frequency acupuncture may be different. Furthermore, the intensity of the stimulation may also play an important role.

In our included studies, most of low-frequency stimulated EA decreased the activity of the LC whereas high frequency increased the activity of the LC, except in the studies of Kwon et al. [26], Lee and Beitz [25], and Qiu et al. [28]. In the studies of Kwon et al. and Lee and Beitz, the low frequency (4 Hz) of EA increased the c-Fos expression in the LC. However, both of these studies used relatively longer stimulation (120 and 180 minutes, resp.) compared to other studies where only 20 to 30 minutes of stimulation were given. Also, in the study of Qiu et al., 2 Hz of acupuncture was administered in a set of four acupoints simultaneously. So, we suppose that strong stimulation such as longer duration of the stimulation or increased number of stimulated acupoints may have led to an incensement of the LC c-Fos expression, as strong stimulation was reported to increase the activity of the LC [44].

Due to the limited numbers of the studies and the differences of the results, in this review, we cannot draw a firm conclusion. More high quality clinical as well as experimental studies are needed, to draw any firm conclusions. Furthermore, research not only in the LC but in other areas of the brain should help the understanding of LC functions, as in the brain different areas are closely related to one another. Also, for the advancement of the research in acupuncture, it might be helpful for researchers of acupuncture to adjust the stimulation criteria (duration, amplitude), so that all researchers could compare the difference of acupoint specific and frequency specific effects of the acupuncture.

## Figures and Tables

**Table 1:** Summary of studies on the effect of acupuncture on the activity of LC neuronal cells.

Authors	Models	EA stimulations			Methods used	Findings
		Acup.	Freq.	Durat.		
*Cao et al. 1983*	*Albino rabbit*	*Ipsilateral LI4, SJ05*	*2-3 Hz*	*20 min.*	*Brain perfusate*	*EA decreased the NE level in perfusate of LC (*156 ± 29* pg/ml to *59.41 ± 7.8* pg/ml; p* < 0.02*). Control group without EA did not have significant change. EA increased the NE level in the perfusate of dorsal horn of the spinal cord. The pain threshold increased from *0.36 ± 0.09* mA to *1.36 ± 0.22* mA *(*p* < 0.001).

Lee and Beitz 1992	SD rat	Bilateral ST36	4 or 100 Hz	180 min	c-Fos expression	Both low (4 Hz) and high (100 Hz) frequency of EA exhibited a significantly greater number of Fos-labeled neurons in the LC. 6.00 ± 2.55 (control); 15.75 ± 4.17 (4 Hz); 13.50 ± 3.07 (100 Hz).

Kwon et al. 2000	SD rat	Bilateral ST36	4 or 100 Hz	120 min.	c-Fos expression	Both low (4 Hz) and high (100 Hz) frequency of EA increased the Fos expression in catecholaminergic neurons in the LC (*p* < 0.05 versus group without EA treatment). Colocalization of FLI neurons and DBH or TH-positive neurons all increased after EA (*p* < 0.05 versusgroup without EA treatment)

*S. Wang and X. Wang 2002*	*L-Dopa-induced hyperactivated bladder/SD rat*	*Bilateral B29 *	*1 Hz*	*30 min.*	*IHC*	*EA decreased the frequent micturition and increased basal bladder pressure in L-dopa injected group (p* < 0.05* versus normal saline injected group).*
						*EA had a significant inhibitory effect on L-dopa-caused feedback increase in the synthesis of DBH in the LC (*15.8 ± 1.28* versus *6.9 ± 1.02*; p* < 0.05*). *

Medeiros et al. 2003	Immobilization stress induced/Albino Wistar rat	Bilateral ST36	100 Hz	60 min.	c-Fos expression	Repeated immobilization (6 days: 1 h/day; 13 days: 2 h/day) significantly reduced the immobilization induced c-Fos expression in LC compared to undisturbed rats (*p* < 0.05). EA increased this decreased level of c-Fos expression in the LC compared to control (1.898 ± 0.38 versus 3.148 ± 0.43; *p* < 0.05).

*Lee et al. 2004*	*Immobilization stress induced/SD rat*	*Bilateral HT3 and PC6 *	*3 HZ*	*30 min.*	*c-Fos expression*	*180 min of immobilization stress produced a significant increase in FLI in the LC (p* < 0.01* versus normal group). EA attenuated immobilization stress induced FLI in the LC compared to control (*20.5 ± 0.8* versus *15.8 ± 1.0*; p* < 0.01*). *

Li et al. 2007	CFA-induced inflammatory pain/SD rat	Bilateral GB30	10 Hz	20 min.	c-Fos expression	EA induced abundant Fos expression in the bilateral LC (110.4 ± 9/per 50 *μ*m section). Untreated naive rats expressed little Fos in this nucleus. Double-immunofluorescence staining demonstrated that Fos was completely colocalized with TH in the LC.

*Wang et al. 2007*	*SD rat*	*MA at the sacral segment (S3) *	*Manual rotation 1.0–1.5 turns/s*	*1 min.*	*Extracellular recording*	*Firing rate of noradrenergic LC neurons decreased significantly from *2.9 ± 1.5* to *1.1 ± 0.8* Hz (p* < 0.001*).*

*Napadow et al. 2009*	*Human*	*Ipsilateral ST36 *	*2 and 15 Hz*	*30 min.*	*fMRI*	*Stimulation with EA deactivated LC activity compared to sham EA (8 cm above the proximal edge of the patella). *

*Park et al. 2013*	*Immobilization stress induced/SD rat*	*Bilateral ST36*	*2 Hz*	*10 min.*	*IHC*	*Immobilization stress induced for 21 days. The TH-immunoreactive expression in the LC was significantly decreased in the ST36 group (p* < 0.001* versus stress induced group).*

Qiu et al. 2015	Two-year-old male goats	GV20, Santai, AH1, and TE8	0, 2, 60, and 100 Hz	30 min	c-Fos and c-Jun expression	c-Fos and c-Jun activity of the LC increased in all three frequencies (2, 60, and 100 Hz) compared to the blank control. Pain threshold was measured using the potassium iontophoresis method. Pain threshold induced by 60 Hz was higher (*p* < 0.01) than that by 0, 2, or 100 Hz.

Hu et al. 2016	One-year-old male goats	GV20, Santai	60 Hz	30 min.	c-Fos expression	Compared with blank control, EA at two sets of acupoints increased c-Fos expression in the LC (9.31 ± 2.39 versus 15.27 ± 2.40; *p* < 0.05).

Italics font style: decreased activity of LC; roman font style: increased activity of LC; Acup., Acupoints; AH1, Ergen; CFA, Complete Freund's Adjuvant; DBH, Dopamine-P-hydroxylase; Durat., duration; EA, electroacupuncture; Freq, frequency; IHC, immunohistochemistry; LC, Locus Coeruleus; L14, Hegu; SJ05, Wai Guan; TE8, Sanyangluo; ST36, Zusanli; FLI, Fos-Like Immunoreactive; fMRI, functional magnetic resonance imaging; GV20, Baihui; GB30, Huan Tiao; TH, tyrosine hydroxylase.
